# The Respirator

**Published:** 1840-10

**Authors:** 


THE RESPIRATOR.

IN an early Number of this Journal, (Vol. III., p. 492,) we gave a favorable report of this ingenious instrument; but as it was then a novelty, we spoke rather anticipately from the evidence of the principle of the instrument, than from any real experience of its value. Three years having passed since then, and the instrument having been during that period in very general use, we are now enabled to speak more positively of its effects. Indeed, these have been observed by so many medical men, and, we doubt not, by the majority of our readers, that any fresh notice of the Respirator may seem almost superfluous; still as there may be some who are not fully acquainted with either its nature or its great value, we deem it our duty, at this season of the year, to call the attention of all our friends to it. In our former notice we gave some account of the construction of the instrument and its principle of action. We will only here repeat that it consists of a series of layers formed of an infinity of fine metallic wires fixed in a frame, and worn over the mouth. All respiration being performed through this, the wires soon acquire considerable warmth from the expired breath, and again yield this up, in part, to the air as it is inspired, so as to temper it ere it reach the lungs. We now speak from our own observation and experience, when we report most favorably of the Respirator. In several instances we have known it productive of the greatest comfort to individuals with irritable air-passages, enabling them to go into the open air in winter without suffering the pain or dyspnœa or cough to which they were otherwise subject in such circumstances; and we have heard of numerous cases of the same kind from our medical and other friends. In consequence of the increased sale of the Respirator, and with a view to its more extensive use, we are happy to find that its inventor, Mr. Jeffreys, to whom pulmonary invalids are deeply indebted, has greatly reduced its price; the instrument being now to be had in most towns at prices varying from ten shillings to twenty or thirty. There are few, if any, practitioners who cannot number one or more patients who, during the winter months, will profit greatly by the use of the Respirator.

